# Rare pathogenic structural variants show potential to enhance prostate cancer germline testing for African men

**DOI:** 10.21203/rs.3.rs-4531885/v1

**Published:** 2024-06-13

**Authors:** Vanessa Hayes, Tingting Gong, Jue Jiang, Riana Bornman, Kazzem Gheybi, Phillip Stricker, Joachim Weischenfeldt, Shingai Mutambirwa

**Affiliations:** University of Sydney; Human Phenome Institute, Fudan University; Garvan Institute of Medical Research; University of Pretoria; University of Sydney; St. Vincent’s Hospital; BRIC, University of Copenhagen; Sefako Makgatho Health Science University

**Keywords:** prostate cancer, pathogenic variants, structural variants, health disparity, African ancestry, germline testing

## Abstract

Prostate cancer (PCa) is highly heritable, with men of African ancestry at greatest risk and associated lethality. Lack of representation in genomic data means germline testing guidelines exclude for African men. Established that structural variations (SVs) are major contributors to human disease and prostate tumourigenesis, their role is under-appreciated in familial and therapeutic testing. Utilising a clinico-methodologically matched African (n = 113) *versus* European (n = 57) deep-sequenced PCa resource, we interrogated 42,966 high-quality germline SVs using a best-fit pathogenicity prediction workflow. We identified 15 potentially pathogenic SVs representing 12.4% African and 7.0% European patients, of which 72% and 86% met germline testing standard-of-care recommendations, respectively. Notable African-specific loss-of-function gene candidates include DNA damage repair *MLH1* and *BARD1* and tumour suppressors *FOXP1*, *WASF1* and *RB1*. Representing only a fraction of the vast African diaspora, this study raises considerations with respect to the contribution of kilo-to-mega-base rare variants to PCa pathogenicity and African associated disparity.

## Introduction

Prostate cancer (PCa) is a significant global health burden and a leading cause of male associated cancer deaths^1^. With one of the highest heritability rates (estimated 58%), PCa risk shows a great degree of variability^2^, particularly when considering a man’s ancestral heritage. In the United States, Black men are at greatest risk for aggressive disease presentation^3^ and depending on age at diagnosis an over double to triple (< 65 years) the risk for PCa-associated mortality than White Americans^4, 5^. Contributed by a complex interaction of socioeconomic factors and genetics^6^, inherited risk includes a combination of both common (low-risk with combined genetic risk scores) and rare (high-risk or pathogenic) germline variants^7, 8^. Revolutionised through advancement of precision oncology, most notably the approval of the poly-(ADP ribose) polymerase (PARP) inhibitors Olaparib^9^ and rucaparib^10^ for the treatment of metastatic castrate resistant PCa for patients harbouring rare pathogenic variants in specified DNA repair genes^11^, has increased the value for germline testing. Furthermore, the National Comprehensive Cancer Network (NCCN) recommends germline testing for all men with metastatic, recurrent or high-risk localized PCa, regardless of family history^12^. Although a significant risk factor for aggressive disease, no consensus could be reached for men of African ancestry^13^, while a recent review further highlighted the knowledge gap^14^.

The lack of consensus for PCa germline testing in Black men is directly attributed to a lack of available data, compounded by a lack of African-relevant genomic data that captures the true extent of elevated genetic diversity. While consensus has yet to be reached for minority inclusion in the benefits of recent breakthroughs in PCa precision oncology, contradictory studies suggest that Black American patients harbour more^15^ and conversely less actionable pathogenic variants than White Americans^16^. The picture is no different for Africa, although more recently PCa genomics has reached the continent with the first whole exome (n = 45 Nigerian)^17^ and whole genome sequencing studies (n = 113 Black South Africans)^18^. Although preliminary, notable differences within Africa are emerging. For example, an elevated frequency of BRCA1 germline mutations reported for Nigerian patients, reflecting African American data^17, 19^, is lacking in Southern African cases^20^. Additionally, we have recently reflected on the lack of the West African exclusive and functionally relevant common PCa susceptibility variants *CHEK2* p.Ile448Ser (rs17886163) and HOXB13 p.Ter285Lys (rs77179853) in Southern Africa^21, 22^. Reporting a 2.1-fold age-adjusted increase in aggressive PCa presentation in Black South African *versus* Black American men^23^, through deep sequenced interrogation for the 20 most common genes included in PCa germline testing panels using NCCN inclusion criteria (Gleason score ≥ 8), we observed a prevalence for rare pathogenic variants of 5.6%^20^, comparable with a single East African study (5.7%)^24^ and almost half that reported for non-Africans (11.8%)^25^. These studies highlight the need for developing African-relevant PCa germline testing panels through African inclusion in genome profiling.

Again, it is well established that Structural Variations (SVs) play a critical role in prostate tumour progression with prognostic and therapeutic potential^26, 27^, including tumours derived from men of African ancestry^18, 28^. Yet, irrespective of patient ancestry, little is known with regards to the contribution of germline potentially pathogenic rare SVs. Typically, greater than 50 bases in length, SVs encompassing large deletions (DEL), duplications (DUP), insertions (INS), inversions (INV) and translocations (TRA), are overlooked and/or difficult to resolve using current germline genetic testing assays. While it is well established that SVs play a critical role in diagnostic screening for inherited genetic diseases^29^, more recently, long-read sequencing has been used to identify potential pathogenic SVs in hereditary cancer syndromes^30^ and known breast cancer susceptibility genes^31^, however, the impact of rare pathogenic SVs on PCa predisposition, and in turn targeted treatment, remains unknown.

Expanding on our earlier work^18, 20, 28^, including deep sequenced germline genomes for 113 African (Black South African) and 57 European (4 South African, 53 Australian) PCa patients, through high-quality SV calling and genotyping, comprehensive gene annotation and best-fit pathogenicity prediction workflow, we interrogate for rare potentially pathogenic SVs (PP-SVs). While agreeably a small study size, this resource is not only unique for the African continent, importantly it provides clinically and technically matched non-African data for direct comparative analyses, while the whole genome approach increases sensitivity for SV detection. As such, the study aims to limit spurious findings between the ancestries, while providing a foundation for further efforts across the continent. Identifying candidate PP-SVs highlights the value of whole genome interrogation not only to improve the detection rate for rare pathogenic PCa variants, but importantly begin to contribute to the much-needed emphasis on an all-African inclusion model for germline testing and associated clinical care.

## Results

### NCCN high-risk characterisation for ancestrally assigned PCa patients

Clinically and technically matched whole genome sequenced germline data (mean coverage 45.9X; range 30.2–97.6X) was derived from 170 PCa patients, ancestrally classified previously using 7,472,833 genome-wide SNVs and population substructure analysis^18^. In brief, 113 Black South African patients presented with an African ancestral genetic fraction of > 85%, while the 57 White patients presented with European ancestral genetic fractions of > 90% (4 South African, 52 Australian) and 73.7% European and 26.3% Asian substructure (1 Australian) (**Supplementary Table 1**). Importantly, although mean age was 5-years younger at presentation or surgery, a greater number of European (86%; 49/57) over African patients (72%; 81/113) met current NCCN guidelines for germline testing based on International Society of Urological Pathology (ISUP) Group Grading defined as high-risk localized PCa (ISUP 4/5 or Gleason score ≥ 8). Notably, we have previously provided evidence for the extension of these criteria for Black South African men to include ISUP 3, which would expand our cohort of high-risk Black men to 82% (93/113)^20^. While Black South Africans present with significantly elevated median and range of prostate specific antigen (PSA) levels (median 244 ng/mL *versus* 9.4), as previously presented^18, 23^, still the study was biased towards over representation of NCCN guidelines for PSA inclusive high-risk PCa for the European (70.2%; 40/57) over African patients (65/113; 57.5%).

### Genome-wide gene-disrupting SV discovery

**Figure 1:**
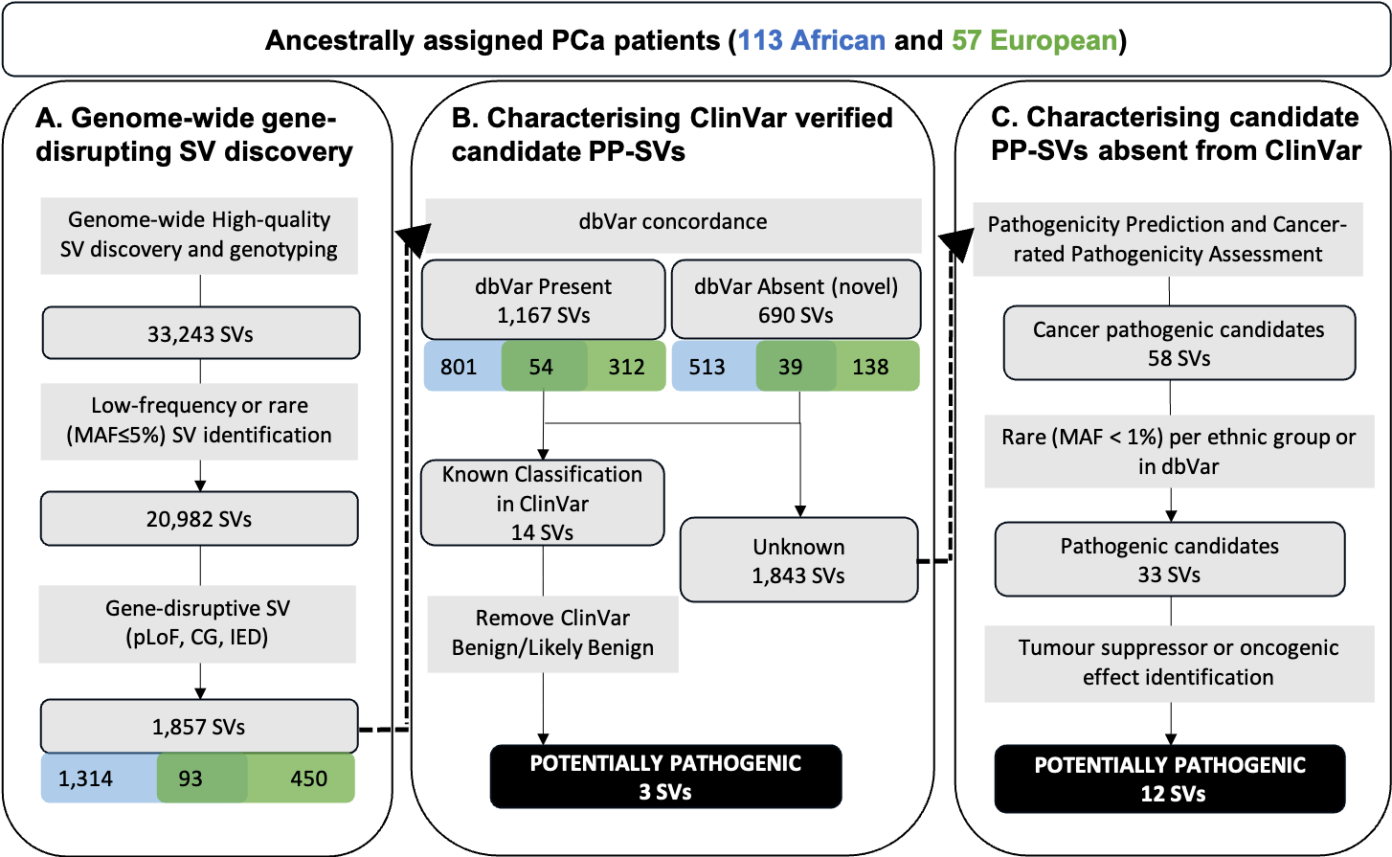
Workflow of PCa potentially pathogenic SV (PP-SV) identification. The detailed criteria to predict the potential pathogenicity were shown in Supplementary Table 3. The identification of tumour suppressor or oncogenic effect for disrupted genes by pathogenic candidates and the related literatures were shown in Supplementary Table 7.

In this study, we identified and genotyped 42,966 high-quality germline SVs. We found a median of 9,206 SVs (range: 8,891 to 9,708) per-African genome, which is significantly higher than the median of 7,490 (range: 7,309 to 8,050) per-European genome (p-value = 1.1e-26 by Wilcoxon test). In total, we identified 38,668 African derived SVs (18,674 private) and 24,292 European derived SVs (4,298 private) (**Supplementary Table 2**). Including only high-quality genotype calls for allele frequency (AF) estimation left a total of 33,243 high-confidence SVs. Excluding for common SVs, defined as minor allele frequency (MAF) > 5%, a total of 20,982 rare (MAF < 1%) and low-frequency (MAF = 1 to 5%) SVs remained across the ancestries for further annotation (Fig. 1).

Further interrogation for gene regions overlapping, we identified 1,857 gene-disruptive SVs, including 1,752 potential Loss-of-Function (pLoF), 52 Copy Gain (CG) and 53 Intragenic Exon DUP (IED) (detailed in Methods). Notably, pLoF, CG and IED SVs can have functional impact on genes through either gene inactivation or increased dosage effect^32^. Conversely, there is no clear or direct coding effect by SVs with other gene impact types, which included in our study 109 partial gene DUP, 22 partial exon DUP, 48 whole-gene INV, 343 promoter SVs, 9,431 intronic SVs and 258 enhancer SVs. As such, the latter SVs were not discussed further. In total, we identified 1,857 (MAF ≤5%) gene-disruptive SVs of which 1,407 are African-relevant, including 93% (1,314) African-private, and 543 European-relevant, including 83% (450) European-private (**Supplementary Table 2**). There were 93 SVs (5%) shared by both African and European PCa patients. The 1,857 gene-disruptive SVs (1,050 rare in both African and European) underwent further downstream interrogation for potential clinical relevance. Of the 1,857 gene-disruptive SVs, 1,167 were previously reported in dbVar database of SVs, while 690 were absent and as such regarded as novel, of which 513 (74%) are uniquely African (Fig. 1).

### Characterising ClinVar verified candidate potentially pathogenic SVs

Of the 1,167 dbVar reported gene-disruptive SVs, 14 (1.2%) were recorded in ClinVar, with three reported as ‘pathogenic’ or ‘likely pathogenic’ based on functional prediction consensus. One 2,958 bp likely pathogenic DEL results in loss of exon 7 in *OCA2* (**Supplementary Fig. 1**), a 5,064 bp pathogenic DEL leads to exon 5–7 loss in *PIGN* (**Supplementary Fig. 2**), while a 235 bp likely pathogenic DUP duplicates exon 3 of *SLC3A1* (**Supplementary Fig. 3**). The *OCA2* and *PIGN* DELs were identified in a single African patient each, while the SLC3A1 DUP presented in two African patients (Table 1).

Although pathogenic in ClinVar, none have been associated with cancer phenotypes and include rather oculocutaneous albinism, multiple congenital anomalies-hypotonia-seizures syndrome and cystinuria, respectively. As such, we searched the literature for plausibility with further ascertainment derived from normal prostate and tumour tissue data sets using GENT2^33^. Reported to be downregulated in numerous cancer types (all-type P < 0.001, GENT2 T-test), although not significant for PCa, pLoF deletion of the pigmentation gene OCA2 has been linked not only to Prader-Willi syndrome, but also Prader-Willi associated malignancies^34^, and melanoma^35^, with recent studies linking melanoma with increased PCa risk^36^. Highly expressed in normal prostate tissue with significant upregulation in tumour tissue (P < 0.001, GENT2 T-test), *PIGN* functions as a cancer chromosomal instability suppressor gene^37, 38^. Although at lower levels, *SLC3A1* is also upregulated in PCa (P < 0.001, GENT2 T-test), with overexpression in breast cancer associated with tumourigenesis^39^. These observations taken together provide the rational for characterising the pLoF *OCA2* and *PIGN* DELs and *SLC3A1* IED as potentially pathogenic SVs (PP-SVs). Notably, all three SVs are reported as rare (irrespective of ancestry) in multiple population-wide studies including gnomAD SV^32^, 1000 genomes Project (1KGP)^40, 41^ and TOPMed SV^42^ (**Supplementary Data 1**).

### Characterising candidate potentially pathogenic SVs absent from ClinVar

Among 1,843 SVs with unknown classification in ClinVar or absent from dbVar, we predicted their potential pathogenicity based on four SV impact prediction tools, including StrVCTVRE^43^, CADD-SV^44^, POSTRE^45^ and PhenoSV^46^. The number of scored SVs by four tools and their types were shown in **Supplementary Fig. 4** and **Supplementary Table 3**. Candidate SVs were required to meet two of the following criteria: StrVCTVRE score ≥0.37, CADD-SV score ≥ 10, POSTRE score ≥0.8 and/or PhenoSV score ≥0.5 (**Supplementary Table 4** and Methods). Based on this criterion, all three ClinVar identified pathogenic or likely pathogenic SVs and the single SV of uncertain significance were successfully annotated as pathogenic candidates, while conversely our workflow excluded for all 10 ClinVar characterised benign SVs (**Supplementary Table 5**). Using our criteria, 291 SVs were defined as PP-SV candidates (107 DELs, 16 DUPs, 11 INVs and 157 TRAs) disrupting 419 genes. In total 190 candidate SVs were private to African and 88 to European patients, with 13 shared between the ancestries (**Supplementary Table 4**).

To further define cancer-related pathogenic potential, we assessed for the presence of disrupted genes by PP-SV candidates in gene sets derived from the Human Molecular Signature Database (MSigDB) oncogenic signature and hallmark gene sets^47^ and COSMIC Cancer Gene Census (COSMIC CGC) cancer driver genes^48^. Requiring disrupted genes in two of the three cancer gene sets, 58 SVs were defined as cancer-related PP-SV candidates, including 20 DELs, 3 DUPs, 6 INVs and 29 TRAs, disrupting 56 genes. Of the 58 candidates, 23 of them were identified with MAF between 1–5% in either African or European patients, leaving 35 rare PP-SV candidates for further consideration, of which 16 have been reported in dbVar. Two dbVar SVs including TRA disrupting gene *NBEA* and *POLR2C* DEL were reported at low-frequencies (AF = 0.03 and 0.01, respectively) (**Supplementary Data 1**) and were therefore excluded from further analysis. Using our criteria, 33 rare cancer-related PP-SV candidates were identified (**Supplementary Data 2** and Fig. 1), including 15 DELs, 3 DUPs (1 IED and 2 CGs), 5 INVs and 10 TRAs.

**Figure 2:**
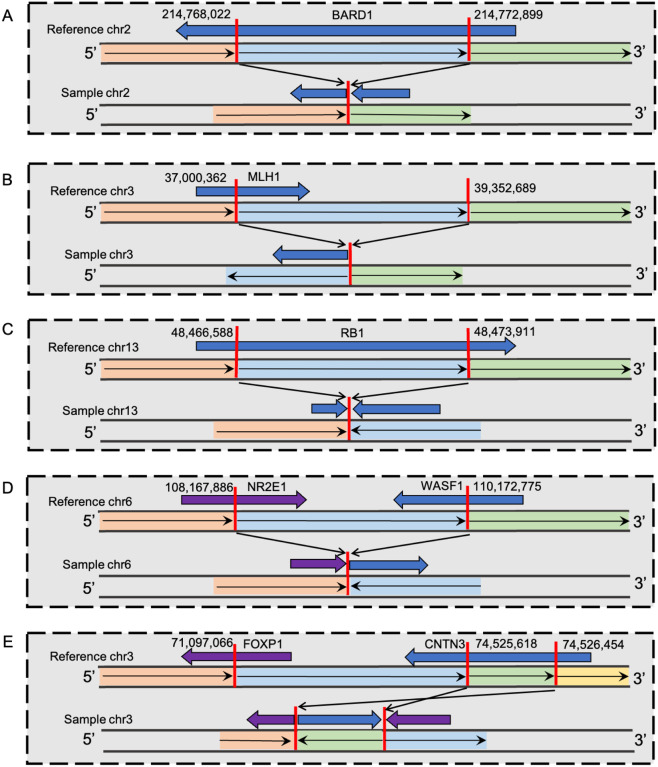
African-specific PP-SVs disrupting well-known pathogenic cancer genes and/or PCa tumour suppressor genes, including DNA damage response genes. (**A**) 4,877 base pLoF deletion on DNA damage repair gene *BARD1*. (**B**) pLoF INV impacting PCa DNA mismatch repair gene *MLH1*. (**C**) pLoF INV impacting PCa tumour suppressor *RB1*. (**D**) pLoF INV impacting PCa tumour suppressor gene *WASF1*. (**E**) pLoF INV impacting PCa tumour suppressor gene *FOXP1*. More details of SV region and/or breakpoints on impacted genes and visual inspection of sequencing reads using Integrative Genomic Viewer^49^ are shown in Supplementary Fig. 8, 12, 13, 14 and 15, respectively.

Of the 15 pLoF DELs, 11 were excluded as PP-SVs, with impacting genes showing oncogenic behaviour in multiple cancer types or no strong evidence for their tumour suppressor effects (**Supplementary Table 7**). Conversely, four pLoF DELs were defined as PP-SVs, impacting known tumour suppressors or established DNA damage repair gene (**Supplementary Table 7**). Two of them are known to dbVar, including a *SLC7A2* 125,146 bp DEL identified in two African (**Supplementary Figs. 5**) and a *DNAJC15* 920 bp DEL in a European patient (**Supplementary Figs. 6**). Another two identified PP-SVs are novel pLoF DELs, which identified in a single African patient each, including a *BCL2L11* 3,275 bp (**Supplementary Fig. 7**) and DNA damage repair gene *BARD1* 4,877 bp DEL (Fig. 2A, **Supplementary Fig. 8**).

Of the two dbVar whole-gene DUPs, the *COL4A2* 339,611 bp CG, with breakpoints disrupting *COL4A1* and *NAXD*, observed in a single African patient is defined as a PP-SV (**Supplementary Fig. 9**), as *COL4A2* indicating oncogenic behaviour in gastric and breast cancers (**Supplementary Table 7**). In contrast, the *TTC27* 703,583 bp DUP observed in a single European patient is afforded ‘cautionary’ PP-SV status (**Supplementary Fig. 10**). Although *TTC27* is absent in three cancer gene databases, the breakpoints disrupt MSigDB and COSMIC CGC genes *BIRC6* and *LTBP1*, resulting in a *LTBP1-BIRC6* gene fusion of unclear effect. Observed in a single European patient, a 3,836 base DUP directly impacts exon 4 of *SLC2A5* (**Supplementary Fig. 11**), which downregulated in PCa (P < 0.001, GENT2 T-test) and has been identified an oncogenic behaviour (**Supplementary Table 7**), therefore allocated PP-SV status.

Of the five pLoF INVs, those impacting *MLH1, RB1* and *WASF1* are in dbVar, while *FOXP1* and *NSD3* INVs are novel. As *NSD3* has been identified as oncogenic in multiple cancers, the associated INV is classified here as unlikely pathogenic, with all remaining pLoF INVs classified as PP-SVs, as they disrupting known to PCa and Lynch Syndrome predisposing DNA mismatch repair gene *MLH1* and PCa tumour suppressor genes *RB1*, *WASF1*, and *FOXP1* (**Supplementary Table 7**). Identified in a single African patient each (**Supplementary Fig. 12–14**), the three dbVar INVs were reported as rare by the recent TOPMed SV study^42^, in which *WASF1* INV was also identified as African-specific (Table 1 and **Supplementary Data 1**). The novel INV impacting *FOXP1* was identified in two African patients (Fig. 2E, **Supplementary Fig. 15**).

Of the 10 pLoF TRAs, five impacting genes of *GRM8, WDR43, NPM1, NUSAP1* and *MECOM* with oncogenic properties (**Supplementary Table 7**), therefore are classified as unlikely pathogenic. *PKHD1* TRA identified in two African patients received a ‘cautionary’ PP-SV classification, as identified potential oncogenic in colon cancer, while potential tumour suppressor in colorectal cancer (**Supplementary Table 7**). As *CTNNA1* was known to have tumour suppressor behaviour across multiple tumour types (**Supplementary Table 7**), here we classify the European-specific pLoF *CTNNA1* TRA as a PP-SV (**Supplementary Fig. 16**). The remaining pLoF TRAs result in *PHC3-PRKACA* (1 African patient), *KCTD3-DST* (2 African patients) and *AK8-DST* (1 European patient, **Supplementary Fig. 17**) novel gene fusions. *PHC3-PRKACA* was classified as ‘cautionary’ PP-SV, as *PHC3* showed potential cancer suppressor effect in PCa, while *PRKACA* appears to portray oncogenic behaviour (**Supplementary Table 7**). Although unknown to PCa, both *DST* and *AK8* have demonstrated tumour suppressor behaviour, conversely, *KCTD3* with an unclear role in cancer (**Supplementary Table 7**). Here we classify *AK8-DST* as a PP-SV, while *KCTD3-DST* is assigned ‘cautionary’ PP-SV status.

### Correlating PP-SVs and ‘cautionary’ PP-SVs with clinical features

The clinicopathological features of the study cohort has been previously described^18, 28^. In brief, African patients show a 5-year greater mean age and 25-fold greater PSA level at diagnosis compared to European patients (**Supplementary Table 1**). Based on our previous observations^20^, high-risk or aggressive PCa were defined as ISUP GG ≥ 3 and conversely, low-risk disease presentation as ISUP GG<3. Biased towards aggressive disease presentation (82% African, 86.0% European), it was notable that all four patients with a pathogenic or likely pathogenic SV presented with aggressive disease at diagnosis, 92.9% (13/14) of PP-SV and 83.3% (5/6) cautionary PP-SV presenting patients (Table 2).

## Discussion

ClinVar defined pathogenic (or likely pathogenic) SVs disrupting *SLC3A1, OCA2* or *PIGN* were observed in 3.5% (4/113) of African patients. Specifically, the *SLC3A1* intragenic exon DUP was identified in two patients presenting with ISUP GG4, while the *OCA2* and *PIGN* pLoF DELs presented in a single patient each with ISUP GG5 and ISUP GG3 PCa, respectively (Table 2). Visually inspecting the three PP-SVs using Integrative Genomic Viewer^49^, *SLC3A1* DUP was found with three supporting read-pairs in sample N0001 (**Supplementary Fig. 3**), and split-reads and more than 40% increase in read depth comparing to ±10 kb of the SV region in both samples (**Supplementary Table 6**), while *OCA2* and *PIGN* DELs were found with 16 and 6 supporting read-pairs respectively (**Supplementary Fig. 1–2**), and have 44–51% reduction in read depth (**Supplementary Table 6**). Solute carrier family 3 member 1 (*SLC3A1*) is an amino acid transporter, which through heterodimerisation with *SLC7A9* is responsible for cystine reabsorption through cationic and neutral amino acid exchange^50^. Mutations, including SVs, in SCL3A1 are associated with cystinuria, an inherited disease that results in the formation of cystine stones in the kidney, with disease presentation suggested to require biallelic loss^51^. *SCL3A1* overexpression has been associated with enhanced tumourigenesis in breast cancer, while blocking *SCL3A1* has suggestive therapeutic potential^39^. *OCA2* is a pigmentation gene with inherited mutations associated with oculocutaneous albinism^52^. Polymorphisms have been associated with skin cancers^53^, as well as clinical response and survival in breast cancer patients having received neoadjuvant chemotherapy^54^. Inherited *PIGN* mutations have been associated with multiple congenital anomalies-hypotonia-seizures syndrome and Fryns syndrome, with some mutations related to milder forms of clinical presentation^55, 56^. Coding for phosphatidylinositol glycan anchor biosynthesis class N, PIGN is involved in the biosynthesis of glycosylphosphatidylinositol, which has been shown to suppress cancer chromosomal instability^37^ through PIGN complexed spindle assembly checkpoint regulation^38^, a common phenomenon in solid tumours^57^. Notably, no previous associations have been made between *SCL3A1, OCA2* or *PIGN* mutation and PCa.

As our study is biased towards under-represented African patients, it is highly plausible that the majority of SVs detected are unlikely to be represented in ClinVar. As such, it is critical that we developed a best-fit workflow for PP-SV prediction. The four SV impact prediction tools used in this study were chosen based on the criteria of easy-to-use (either web-based or packed as software), providing pathogenicity scores or labels, accepting multiple SVs and covering all SV types. However, there are multiple factors to be taken into consideration when using SV impact prediction tools to establish potential pathogenicity, as different tools have limitations in applicable SV types, regions or diseases, as well as different scoring systems. While all tools can predict the impact of DELs and DUPs, StrVCTVRE is limited to DELs and DUPs in exonic regions. Besides predicting the simpler SVs, CADD-SV is capable of annotating INSs and POSTRE annotates INVs and TRAs, while PhenoSV is able to predict the impact of all these three types. POSTRE doesn’t work for all diseases or phenotypes. Therefore, combining multiple tools is necessary to cover all SV types and increase the confidence level. Another factor is the choice of threshold to establish pathogenicity. POSTRE and PhenoSV defines the threshold of pathogenicity, but StrVCTVRE and CADD-SV are limited to scores and calling for thresholds to be established depending on individual study aims. In this study, we have decided the thresholds based on tools’ validated results from database (90% sensitivity in ClinVar by StrVCTVRE^43^ and top 10% in gnomAD by CADD-SV^44^). When combining results from multiple tools, we found the requirement of passing thresholds of all four tools identified two PP-SV candidates (out of 1,843 SVs) (**Supplementary Table 4**), with notable failure to identify the three ClinVar pathogenic/likely pathogenic SVs (**Supplementary Table 5**). As such, PP-SV candidate classification in this study required an SV to pass thresholds of at least two impact prediction tools, with disrupted genes requiring further clarification as hallmark or drivers in cancer gene databases (MSigDB and COSMIC CGC).

Using our described workflow, 12 SVs were predicted as PP-SVs, identified in 7.0% (4/57) of European and 8.8% (10/113) of African patients, bringing the total of African patients presenting with a potential pathogenic SV to 12.4% (14/113). Remarkably, five of our African-specific PP-SVs included well-known pathogenic cancer genes and/or PCa tumour suppressor genes, including DNA damage response genes. Most notably, the DNA mismatch repair tumour suppressor gene *MLH1* commonly mutated in Lynch Syndrome, including cases with PCa^58^, is a known candidate gene in PCa germline testing panels^20^. While PCa patients presenting with pathogenic *MLH1* mutations were reported to have significantly higher disease burden for African Americans^24^, here we found a dbVar known *MLH1* pLoF INV with around 11 supporting short read-pairs (**Supplementary Fig. 12**) in a 64 year old African male presenting with ISUP GG4 at diagnosis. Not recognised as a PCa germline testing panel gene, *FOXP1* is an established PCa tumour suppressor driver gene, with CN loss increasing cell proliferation and migration, and poor prognosis^59^. Recently, we showed *FOXP1* to be equally impacted by predominantly CN loss in African compared with European derived tumours (20% of 183 tumours)^18^. Here we found a germline inverted duplication impacting *FOXP1* with around 18 supporting read-pairs in two African patients (**Supplementary Fig. 15**). Notably, one African patient (UP2101) presented 10 years earlier than the cohort average receiving an ISUP GG5 diagnosis. Loss of the *BRAC1* associated RING domain-1 (*BARD1*) DNA damage repair gene has been found to induce homologous recombination deficiency and increase the sensitivity to PARP inhibitor in PCa cell lines^60^. Here the novel *BARD1* exon 5 DEL, supported by 10 read-pairs and with around 50% reduction in read depth comparing to ±10 kb of the DEL region (**Supplementary Fig. 8, Supplementary Table 6**), was identified in a 62-year-old African PCa patient with unknown pathology. While a paediatric cancer predisposing tumour suppressor gene commonly mutated in retinoblastoma and to a lesser extent osteosarcoma^61^, and less common as an adult cancer predisposing gene^62^, *RB1* is recognised as one of five most prevalent somatically mutated genes in metastatic cancers^63^, with *RB1* loss in prostate tumours associated with poor patient outcomes^64^. To the best of our knowledge, this is the first report of a germline potentially pathogenic *RB1* PCa variant, which includes a pLoF INV of exon 24 with three supporting read-pairs (**Supplementary Fig. 13**) in a single ISUP GG3 diagnosed African patient. Lastly, the tumour suppressor gene *WASF1* with loss associated with aggressive or metastatic lethal PCa^65^. Identifying a potentially pathogenic INV previously reported at MAF of 9.6e-05 in Africans and resulting in *NR2E1-WASF1* fusion was identified in a single African patient presenting at 70 years of age with ISUP GG5 PCa, showed 14 supporting read-pairs (**Supplementary Fig. 14**).

Other notable PP-SV DELs impacting tumour suppressor genes unknown to PCa, includes *SLC7A2* and *DNAJC15*. Knockdown of *SLC7A2* has been shown to promote viability, invasion and migration of ovarian cancer^66^ and enhance proliferation of non-small-cell lung cancer cells^67^, while *DNAJC15* has tumour suppressor behaviour in breast cancer^68^. Identified in two African patients presenting with ISUP GG5 disease, loss of *SLC7A2* exons 1 and 2, supported by 10 read-pairs and with around 50% reduction in read depth (**Supplementary Fig. 5 and Supplementary Table 6**) has previously been reported in African populations at MAF of 0.03 (**Supplementary Data 1**). Specific to Europeans (MAF = 1.0e-04), loss of *DNAJC5* exon 4 supported by 13 read-pairs and with around 50% reduction in read depth (**Supplementary Fig. 6 and Supplementary Table 6**), was identified in a single European patient presenting for surgery at age 63 years with ISUP GG5 disease. While not associated with PCa, the loss of *BCL2L11* and *CTNNA1* has been identified to leading tumourigenesis and promoting invasion and metastasis of multiple cancers^69, 70^. Here the novel *BCL2L11* pLoF DEL on exon 2 with more than 20 supporting read-pairs and with around 50% reduction in read depth (**Supplementary Fig. 7 and Supplementary Table 6**) was identified in a single African patient presenting at age 71 years with ISUP GG5 PCa, while the novel pLoF TRA interrupting *CTNNA1* with more than 20 supporting read-pairs (**Supplementary Fig. 16**) in a single European patient presenting at age 59 years with ISUP GG5 PCa. Another identified novel potentially pathogenic inter-chromosomal TRA with around 18 supporting read-pairs (**Supplementary Fig. 17**) leading to a *AK8-DST* fusion in a single European patient (ISUP GG1, 67 years). Although no associations have been made between PCa, higher expression of *DST* has been identified to promote pathogenesis and development of breast cancer, while *AK8* downregulation has been found to promote migration and invasion of uterine carcinosarcoma^71^.

Two known PP-SVs identified to potentially increase gene dosage of well-known oncogenes *COL4A2* and *SLC2A5*, through whole-gene duplication and intra-genic exon duplication respectively. Although not associated with PCa, *COL4A2* loss has been identified to inhibit triple-negative breast cancer cell proliferation and migration^72^ and its mutations as risk factor for familial cerebrovascular disease^73^, while inactivation of *SLC2A5* has been found to inhibit cell proliferation and migration in multiple cancer cell lines^74^. The whole *COL4A2* DUP with more than 20 supporting read-pairs and more than 50% gain in read depth (**Supplementary Fig. 9** and **Supplementary Table 6**) was identified in a single African patient (ISUP GG4, 71 years) and the exon 4 DUP in *SLC2A5* with more than 20 supporting read-pairs and more than 50% increase in read depth (**Supplementary Fig. 11** and **Supplementary Table 6**) was identified in a single European patient (ISUP GG5, 70 years).

Using short-read sequencing data for SV calling and genotyping remains a potential limitation, appreciating that SVs in difficult-to-sequence regions may have been overlooked^75^. To ensure the highest possible accuracy of SV detection and population allele frequency estimation, we required high-confidence calls from two SV callers and high-quality genotype calls at both the population- and individual-level, while all PP-SVs were visually inspected. Due to lack of available expression data, we were unable to validate the direct impact of identified PP-SVs and cautionary PP-SVs. Further guidelines related to criteria for pathogenic SV identification using short read sequencing technologies and/or long read sequencing approaches are required, making these methods accessible for routine germline testing.

## Conclusion

Here we have described a first-of-its-kind pathogenicity investigation of SVs in PCa patients with ancestry disparity. We observed three ClinVardefined pathogenic or likely pathogenic PP-SVs (*SLC3A1, OCA2* and *PIGN*) and 12 predicted PP-SVs, including seven known SVs (*SLC7A2, DNAJC15, COL4A2, SLC2A5, WASF1, MLH1* and *RB1*), and five novel SVs (*BCL2L11, BARD1, FOXP1, CTNNA1* and *AK8-DST*), suggesting that inherited SVs may constitute an under-appreciated contribution to PCa pathogenicity. Furthermore, the identification of African-private (eight known and three novel) and European-private (two known and two novel) PP-SVs allows for further speculation with regards to associated racial disparities, while improving the detection rate for PCa germline testing with SV inclusivity, and in turn raising limitations for African inclusion and associated clinical care.

## Methods

### WGS data generation

To avoid technical and analytical biases, all samples (whole blood) were processed (beginning at DNA extraction), data generated and analysed within a single laboratory using a single computational pipeline, as previously described^18, 28^. In brief, whole-genome sequencing data were generated using Illumina HiSeq X Ten (21 cases) or NovoSeq (149 cases) instruments with 2×150 cycle paired-end mode at the Kinghorn Centre for Clinical Genomics (Garvan Institute of Medical Research, Australia). Following the BROAD’s best practice recommendations for “data pre-processing for variant discovery”, sequencing reads were aligned to GRCh38 reference genome with alternative contigs using scalable FASTQ-to-BAM (v2.0) workflow with default settings^76^. The mean depth of coverage for all samples were 45.9X (range 30.2–97.6X).

### Structural variant calling and high-confidence SV filtering

Germline SVs were called using Manta (v1.6.0)^77^ and GRIDSS (v2.13.3)^78, 79^. SV types reported by Manta included DEL, tandem DUP, INS and adjacent breakends (BNDs) for a fusion junction with inverted sequence or in an inter-chromosomal rearranged genome. Pairs of BND in inverted junction were annotated as inversions (INV). Pairs of BND in different chromosomes were annotated as inter-chromosomal translocations (TRA). Conversely, GRIDSS reports BND for all fusion junctions resulting from any SV event. Simple SV types, defined as DEL, DUP, INS, INV and TRA, were assigned based on the strands and ALT field in VCF (modified from GRIDSS accompanied R script: simple-event-annotation.R).To obtain high-confidence SV call set, we integrated call sets from Manta and GRIDSS and generated concordant call set for each genome. Two SV calls were considered as concordant if they were reported as “PASS” by one of the two callers and have matching SV type and reported breakpoint positions within 200bp of each other. *Bedtools pairtopair*^80^ was used to compare two call sets.

### Population-level genotyping and high-confidence genotype call filtering

We used Graphtyper2 (v2.7.5)^81^ to re-genotype SVs for all samples. Following published guidelines, we merged all high-confidence SV set per-sample (individual VCFs) using svimmer (https://github.com/DecodeGenetics/svimmer) with default parameters. The individual VCFs were in format of Manta VCFs, as Manta provides detailed information on the exact breakpoint sequence, which is the essential information required by Graphtyper2. We extracted all SVs with “aggregate” model as suggested, and obtained 57,096 SVs with “PASS” in FILTER field in VCF. To further filtering SV genotype calls on a per-sample basis, we required more than 50% genotype calls as “PASS” (PASS_ratio ≥ 0.5 in INFO field), resulting in 42,966 SVs.

To further filtering SV genotype calls on a per-sample basis, we set SV genotype as missing if genotype filter tag (FT) is not “PASS” for all SVs, except BND. For BND, as FT tag is not available, we set BND genotype with genotype quality (GQ) < 20 as missing. We then excluded SVs with genotype missingness rate > 20% in either African or European genomes, resulting in 33,340 SVs. We further removed 97 SVs with allele frequency of 100%, indicating the difference of sample genomes to reference genome. The allele frequency of each SV was then calculated based on the high-quality genotype calls only.

### Gene annotation and functional impact of SVs

All SVs were annotated against gene regions from the Ensembl human gene annotation file (GRCh38 assembly, release 108). As multiple transcripts can be available for a single gene, the Ensembl Canonical transcript was used (http://www.ensembl.org/info/genome/genebuild/canonical.html). By comparing the position of SV breakpoint with gene regions using bedtools^80^, we examined nine gene overlapping categories with gnomAD^32^, including potential Loss of Function (pLoF), Copy Gain (CG), Intragenic Exon DUP (IED), partial gene DUP, whole-gene INV, UTR SVs, promoter SVs, intronic SVs and intergenic SVs. In addition, we defined partial-exon DUP as both breakpoints contained within the same gene, while neither both within exons (pLoF) nor fully overlapped at least one exon (IED). Promoters were defined as 1kb window before each transcription start site on the transcribed strand. We labelled SVs as enhancer-disruptive if at least one breakpoint was contained within a gene’s enhancer, by comparing to GeneHancer^82^ regulatory elements regions. GeneHancer regulatory elements and gene interactions “double elite” subset was downloaded from UCSC Table *geneHancerInteractionsDoubleElite* [last updated 15/01/2019] from GeneHancer track for GRCh38. The transcript structure plots were generated based on Ensembl human gene annotation (GRCh38 assembly, release 108) using R package ggtranscript (v0.99.3)^83^. The sequencing depth of DEL or DUP regions and their ±10 kb regions were calculated using samtools (v1.6) *depth* command^84^.

Short-read data detect the SV signatures from aligned reads around the SV breakpoints and is hard to capture the whole large SVs^85^. Therefore, we restricted the disrupted genes of SVs greater than 1Mbp to be genes overlapped by SV breakpoints for downstream analysis.

### Identification of dbVar concordance and novel SVs

The NCBI’s database of human genomic structural variation (dbVar) [last updated 30/10/2023]^86^ were used to identify dbVar concordance and novel SVs. The dbVar database included a total of 6,476,337 unique SVs, including 86,686 SVs with interpretations of their significance to disease in ClinVar database^87^. Structural variants concordant to dbVar SVs were defined as having both breakpoints within 200 bases of dbVar defined SV breakpoints. The ancestry related variant allele frequency of SVs (**Supplementary Data 1**) were derived from dbVar pages of SVs or VCFs uploaded by different dbVar studies to dbVar’s FTP site.

### Pathogenicity prediction

The pathogenicity of SVs were predicted through prediction tools StrVCTVRE^43^, CADD-SV^44^, POSTRE^45^ and PhenoSV^46^. StrVCTVRE only scores the deleteriousness of DEL and DUP overlapping one or more exons, CADD-SV scores DEL, DUP and INS, POSTRE predicts the impact of DEL, DUP, INV and TRA, and PhenoSV works for all five SV types. As POSTRE only accepts genome coordinates on reference genome Hg19, the *liftOver* function from *rtracklayer* package in R was used to lift SV coordinates from Hg38 to Hg19. As suggested by StrVCTVRE, the ClinVar 90% sensitivity threshold (0.37) was used to define potentially pathogenic SVs. The scaled CADD-SV scores range from 0 (potentially benign) to 48 (potentially pathogenic), indicating the position of the input SV within the gnomAD-SV score distribution. The threshold of 10 for CADD-SV score was used to establish potential pathogenicity, corresponding to top 10% score observed in gnomAD-SV. The threshold of 0.8 and 0.5 for POSTRE and PhenoSV score respectively was used in this study, which is the threshold of pathogenicity labelling defined by POSTRE and PhenoSV.

The hallmark gene sets and oncogenic signature gene sets were downloaded from the Human Molecular Signature Database (MSigDB v2023.1)^47^. The MSigDB oncogenic signature gene sets included genes representing signatures of cellular pathways which are often dis-regulated in cancer. Cancer-driver genes were downloaded from COSMIC Cancer Gene Census (GRCh38 COSMIC v98, downloaded 26/09/2023).

## Figures and Tables

**Table 1:** Candidate potentially pathogenic (PP) SVs identified in 170 PCa patients.

Genes	Gene impact type^1^	chrom1	pos1	chrom2	pos2	SV type	ClinVar / dbVar concordance	MAF African (this study)	MAF European (this study)	MAF African (dbVar)^2^	MAF European (dbVar)^2^
**Potentially Pathogenic SV (PP-SV)**											
*SLC3A1*	IED	chr2	44281377	chr2	44281612	DUP	Likely pathogenic	0.01^3^	0	0.0075	1.3e-04
*OCA2*	pLoF	chr15	28017719	chr15	28020677	DEL	Likely pathogenic	0.004	0	0.0015	0.001
*PIGN*	pLoF	chr18	62152637	chr18	62157701	DEL	Pathogenic	0.004	0	0.0013	1.3e-04
*SLC7A2*	pLoF	chr8	17418976	chr8	17544122	DEL	In dbVar	0.009	0	0.003	0
*DNAJC15*	pLoF	chr13	43078470	chr13	43079390	DEL	In dbVar	0	0.009	0	1.0e-04
*BCL2L11*	pLoF	chr2	111122626	chr2	111125901	DEL	novel	0.005	0	NA	NA
*BARD1*	pLoF	chr2	214768022	chr2	214772899	DEL	novel	0.005	0	NA	NA
*COL4A2/COL4A1*	CG	chr13	110294204	chr13	110633815	DUP	In dbVar	0.005	0	1.3e-04	6.3e-06
*SLC2A5*	IED	chr1	9045605	chr1	9049441	DUP	In dbVar	0	0.009	7.3e-04	0.002
*FOXP1*	pLoF	chr3	71097066	chr3	74525618	INV	novel	0.009	0	NA	NA
*WASF1*	pLoF	chr6	108167886	chr6	110172775	INV	In dbVar	0.004	0	9.6e-05	0
*MLH1*	pLoF	chr3	37000362	chr3	39352689	INV	In dbVar	0.004	0	4e-04	6.4e-06
*RB1*	pLoF	chr13	48466588	chr13	48473911	INV	In dbVar	0.004	0	1.8e-04	1.3e-05
*CTNNA1*	pLoF	chr5	138903881	chr19	21614900	TRA	novel	0	0.009	NA	NA
*AK8-DST*	pLoF	chr9	132876361	chr6	56896165	TRA	novel	0	0.009	NA	NA
**PP-SV candidates classified as ‘cautionary’**											
*LTBP1/BIRC6*	CG	chr2	32403832	chr2	33107415	DUP	In dbVar	0	0.009	1.0e-04	0.0018
*PHC3-PRKACA*	pLoF	chr3	170090742	chr19	14110142	TRA	novel	0.004	0	NA	NA
*KCTD3-DST*	pLoF	chr1	215567414	chr6	56652607	TRA	novel	0.009	0	NA	NA
*PKHD1*	pLoF	chr6	51981375	chr15	30874073	TRA	novel	0.009	0	NA	NA

1 Gene impact type based on gene annotation. pLoF: Potential loss-of-function. CG: Copy gain. IED: Intragenic Exon Duplication.

2 The ancestry related MAF in dbVar were based on gnomAD^32^ or TOPMed^42^ SV study. The detail of all dbVar studies (dbVar study name and ID) and reported allele frequencies were shown in **Supplementary Data 1**.

3 Presenting at low-frequency rather than rare variants within the ancestrally-defined patient cohort.

**Table 2:** Clinicopathological features of patients by ethnicity presenting with potentially pathogenic (PP) SVs and cautionary PP-SVs as defined by this study criteria.

Gene name	Pathogenicity	SV type	Patient ID	Ethnicity	Age	PSA	ISUP GG	Family history
*SLC3A1*	PP-SV Likely Pathogenic	DUP	N0001	African	75	22.9	4	
			SMU094	African	64	15	4	
*OCA2*	PP-SV Likely Pathogenic	DEL	N0059	African	79	153	5	
*PIGN*	PP-SV Pathogenic	DEL	SMU083	African	86	40.5	3	
*SLC7A2*	PP-SV	DEL	UP2035	African	70	680	5	
			KAL0054	African	64	42.9	5	
*DNAJC15*	PP-SV	DEL	17135	European	63	7.8	5	
*BCL2L11*	PP-SV	DEL	KAL0101	African	71	32.3	5	
*BARD1*	PP-SV	DEL	N0073	African	62	unknown	unknown	
*COL4A2/COL4A1*	PP-SV	DUP	UP2039	African	71	319	4	
*SLC2A5*	PP-SV	DUP	11099	European	70	9.9	5	
*FOXP1*	PP-SV	INV	UP2101	African	57	75	5	
			N0084	African	65	591	4	
*WASF1*	PP-SV	INV	N0048	African	70	83.3	5	
*MLH1*	PP-SV	INV	SMU080	African	64	23.3	4	Sister with cervical cancer
*RB1*	PP-SV	INV	SMU064	African	70	13.7	3	
*CTNNA1*	PP-SV	TRA	13179	European	59	8.4	5	
*AK8-DST*	PP-SV	TRA	11452	European	67	11	1	
*LTBP1/BIRC6*	Cautionary PP-SV	DUP	5287	European	54	4.3	5	
*PHC3-PRKACA*	Cautionary PP-SV	TRA	SMU061	African	65	12.1	3	Mother with stomach cancer
*KCTD3-DST*	Cautionary PP-SV	TRA	UP2039	African	71	319	4	
			SMU101	African	70	4.3	3	
*PKHD1*	Cautionary PP-SV	TRA	N0056	African	70	153	5	
			SMU196	African	47	9.5	1	

## Data Availability

The sequence data were obtained and accessible through Jaratlerdsiri et al^18^, in the European Genome-Phenome Archive (EGA; https://ega-archive.org) under overarching accession EGAS00001006425 and including the Southern African Prostate Cancer Study (SAPCS) Dataset (EGAD00001009067) and Garvan/St Vincent’s Prostate Cancer Database (EGAD00001009066). The dbVar SV sites and their variant allele frequencies were downloaded from https://www.ncbi.nlm.nih.gov/dbvar. The ENSEMBL gene set was downloaded from https://www.ensembl.org. The MSigDB gene sets are available at https://www.gsea-msigdb.org/gsea/msigdb/human/collections.jsp#H. The gene set in COCMIC CGC was downloaded from https://cancer.sanger.ac.uk/census. The scripts for sequence read alignment and quality control are available at GitHub (https://github.com/Sydney-Informatics-Hub/Bioinformatics). All computational code for SV callset comparison and integration are available at GitHub (https://github.com/tgong1/StructuralVariantUtil)^88^.

## Abbreviations

PCa: Prostate cancer
SV: Structural variation
DEL: Deletion
INS: Insertion
DUP: Duplication
INV: Inversion
TRA: Translocation
WGS: Whole genome sequencing
PSA: Prostate Specific Antigen
BND: Breakend
ISUP GG: International Society of Urological pathology Group Grading
pLoF: potential Loss of Function
CG: Copy Gain
IED: Intragenic Exon Duplication
PPSV: potentially pathogenic SV
NCCN: National Comprehensive Cancer Network
1KGP: 1000 genomes Project Phase3
COSMIC CGC: COSMIC Cancer Gene Census
